# The clinical characteristics and outcomes of patients with systemic sclerosis with myocardial involvement

**DOI:** 10.1186/s13023-023-02699-1

**Published:** 2023-04-14

**Authors:** Huilin He, Jinzhi Lai, Jiaxin Zhou, Yong Hou, Dong Xu, Mengtao Li, Xiaofeng Zeng

**Affiliations:** 1 Department of Rheumatology and Clinical Immunology, Peking Union Medical College Hospital (PUMCH), Chinese Academy of Medical Sciences, National Clinical Research Center for Dermatologic and Immunologic Diseases (NCRC-DID), Ministry of Science & Technology; State Key Laboratory of Complex Severe and Rare Diseases; Key Laboratory of Rheumatology and Clinical Immunology, Ministry of Education, No.1 Shuaifuyuan Wangfujing Dongcheng District, Beijing, 100730 China; 2 Department of Cardiology, Peking Union Medical College Hospital, Beijing, China

**Keywords:** Systemic sclerosis, Myocardial involvement, Clinical features, Treatment, Follow-up

## Abstract

**Background:**

Myocardial involvement (MI) is the primary cause of death in patients with systemic sclerosis (SSc). We analyzed patients with SSc and MI to identify their characteristics and outcome.

**Method:**

We retrospectively collected data from SSc patients with MI admitted to Peking Union Medical College Hospital between January 2012 and May 2021. SSc patients without MI were randomly selected as controls after matching age and gender at a ratio of 1:3.

**Results:**

In total, 21 SSc patients (17 females) with MI were enrolled. The mean age at SSc onset was 42.3 ± 15.1 years old. Compared with controls, myositis (42.9% vs. 14.3%, P = 0.014) and elevation of CK (33.3% vs. 4.8%, P = 0.002) were more common in patients with MI. Of the 7 patients without cardiovascular symptoms, 3 /5 showed elevations in cardiac troponin-I (cTnI), 6 showed elevations of N-terminal brain natriuretic peptide (NT-proBNP). Eleven patients were followed up for a median period of 15.5 months and four patients developed newly occurring left ventricular ejection fraction (LVEF) < 50%.

**Conclusion:**

One third of SSc patients with MI were asymptomatic. Regular monitoring of CTnI, NT-proBNP and echocardiography is helpful for the diagnosis of MI during the early stages. Its prognosis is poor.

## Introduction

Systemic sclerosis (SSc) is an autoimmune disease of unknown etiology that is characterized by abnormal immune activation, neovascularization and vascular remodeling, eventually leading to the tissue fibrosis, affecting the skin and various internal organs [1]. SSc is an orphan disease with an estimated global incidence of 0.1‰ [2] and often involves multiple organ systems, as demonstrated by the frequent occurrence of gastroesophageal reflux, interstitial lung disease and cardiomyopathy [3]. Myocardial lesions and cardiovascular disease are both significant causes of death in SSc patients [4]. Overt clinical symptoms of heart disease consistently indicate irreversible changes in the structure and function of the heart. Therefore, it is important to perform early diagnosis and interventions in SSc patients with cardiac complications, so we could reduce irreversible structural changes of the heart and prolong the survival time.

This study aimed to use doppler echocardiography and/or cardiac magnetic resonance (CMR) to investigate the characteristics and outcomes of SSc patients with myocardial involvement (MI).

## Methods

### Study population

Data were retrospectively collected from consecutive SSc patients with myocardial involvement (MI) who had been admitted to Peking Union Medical College Hospital (PUMCH) between January 2012 and May 2021. All patients fulfilled the 2013 American College of Rheumatology/European League Against Rheumatism (ACR/EULAR) classification criteria for SSc [5]. Patients were divided into two groups: diffuse cutaneous SSc (dcSSc) and limited cutaneous SSc (lcSSc). DcSSc was defined as skin thickening extending proximally to the elbows and knees or including the trunk. LcSSc involved skin sclerosis that was confined distally to the elbows and knees or face.

MI was defined as one of the following: (1) evidence of myocardial damage (inflammation or fibrosis) by cardiac magnetic resonance (CMR) imaging; or (2) a left ventricular ejection fraction (LVEF) < 50% by echocardiography [6]. Myocarditis and myocardial infarction caused by other diseases were excluded. Other causes of myocardial lesions, such as coronary atherosclerosis, viral infections, and thyroid dysfunctions, were also excluded. SSc patients without MI over the same period were randomly selected as controls after matching for age (± 3 year) and sex at a ratio of 1:3.

### Data collection

Clinical characteristics, laboratory test results, imaging examinations and treatment data were collected when patients were admitted to PUMCH. All patients underwent clinical (i.e., history and physical examination including cardiovascular symptoms and signs) and instrumental evaluations (including routine blood tests, urine examinations, biochemical examination, creatine kinase (CK), erythrocyte sedimentation rate (ESR), C-reactive protein (CRP), immunoglobulin (Ig), antinuclear antibody test, 12-lead electrocardiogram (ECG), echocardiography (UCG). Some patients who had abnormal clinical or instrumental cardiac findings were further evaluated by CMR or cardiac radionuclide examinations. Interstitial lung disease (ILD) was defined as ground glass opacification or fibrosis on high-resolution computed tomographic (HRCT) imaging. Pulmonary arterial hypertension (PAH) was defined as a mean pulmonary arterial pressure (mPAP) ≥ 25 mmHg, a pulmonary artery wedge pressure (PAWP) ≤ 15 mmHg and a pulmonary vascular resistance (PVR) > 3 Wood units by right heart catheterization (RHC) or an estimated systolic pulmonary artery pressure (SPAP) ≥ 40 mmHg on echocardiography when RHC was unavailable. Leukopenia was defined as a white blood cell (WBC) count < 3.5 × 10^9^/L while thrombocytopenia was defined as a platelet count < 100 × 10^9^/L, excluding other causes, such as drug and infection. We performed a follow-up of SSc patients with MI based on outpatient medical records or admission records.

### Statistical analysis

Data were analyzed using SPSS 26.0 (Statistical Package for the Social Sciences, IBM Corp., Armonk, NY, USA). Quantitative variables are presented as mean ± standard deviation on normal distribution or medians (interquartile range, IQR) on non-normal distribution. Qualitative data are presented as the number of cases and percentage. Differences between groups were analyzed by analysis of variance (ANOVA), the Mann-Whitney test or the Chi-squared (χ2) test, depending on the distribution of the variables. P values < 0.05 were considered to be statistically significant.

## Results

### Demographic features

In total, 21 SSc patients with MI were included in this study; most were females (17, 81%). The mean age of SSc onset and diagnosis were 42.3 ± 15.1 years and 43.6 ± 15.3 years, respectively. The age of MI diagnosis was 46.6 ± 15.8 years (Table 1).

**Table 1:** Demographic characteristics of SSc patients with and without myocardial involvement

	Myocardial involvement (n = 21)	Non- myocardial involvement (n = 63)	P value
Age at SSc onset (years)	42.3 ± 15.1	38.1 ± 13.6	0.239
Age at SSc diagnosis(years)	43.6 ± 15.3	42.8 ± 14	0.824
Age at MI dianosis (years)	46.6 ± 15.8	-	-
Gender/Male (%)	4 (19)	12 (19)	1
BMI	22.0 ± 5.0	22.0 ± 3.6	0.939
Hypertension	3 (14.3)	5 (7.9)	0.668
Diabetes	1 (4.8)	1 (1.6)	0.440
Smoking	2 (9.5)	3 (3.6)	0.790

MI: myocardial involvement; BMI: body mass index; dcSSc: diffuse cutaneous SSc

### Clinical/laboratory/ imaging features

Patients with MI had more diffuse cutaneous SSc (dcSSc) when compared with patients without MI (71.4% vs. 49.2%, P = 0.076) numerically. Compared with SSc patients without MI, SSc patients with MI had a significantly reduced incidence of puffy fingers (19% vs. 50.8%, P = 0.011), gastroesophageal reflux (23.8% vs. 54%, P = 0.016), but a significantly higher incidence of myositis (42.9% vs. 14.3%, P = 0.014) and hydropericardium (42.9% vs. 11.1%, P = 0.004) (all P < 0.05) (Table 2). Elevated CK (33.3% vs. 4.8%, P = 0.002) and anti-SCL70antibody (71.4% vs. 36.3%, P = 0.005) positivity were statistically higher in SSc patients with MI (Table 3). No significant difference was found in pulmonary function test between two groups (all P>0.05).

**Table 2:** The clinical manifestations of SSc patients with and without myocardial involvement

	Myocardial involvement (n = 21)	Non- myocardial involvement (n = 63)	P value
Subset (dcSSc)	15 (71.4)	31 (49.2)	0.076
Overlap syndrome	1 (4.8)	11 (17.5)	0.280
Puffy fingers	4 (19)	32 (50.8)	0.011
Raynaud’s phenomenon	19 (90.5)	61 (96.8)	0.259
Telangiectasia	5 (23.8)	24 (38.1)	0.233
Loss of finger pad substance	3 (14.3)	22 (34.9)	0.073
Digital ulcers	9 (42.9)	18 (28.6)	0.225
Gangrene	3 (14.3)	2 (3.2)	0.183
Arthritis/ arthralgia	5 (23.8)	23 (36.5)	0.285
Myositis	9 (42.9)	9 (14.3)	0.014
Gastroesophageal reflux	5 (23.8)	34 (54)	0.016
ILD	15 (71.4)	47/58 (81)	0.543
PAH	2 (9.5)	19 (30.2)	0.059
Hydropericardium	9 (42.9)	7 (11.1)	0.004
LV diastolic dysfunction	4 (19)	7 (11.1)	0.575
Scleroderma renal crisis	1 (4.8)	1 (1.6)	0.440
Proteinuria	6 (23.1)	6/60 (8.0)	0.090
Modified Rodnan skin score	10 (1.5,33.3) /10	4 (2.5,8) /62	0.134

ILD: Interstitial lung disease; PAH: Pulmonary arterial hypertension LV: left ventricular; EF: Ejection fraction;

**Table 3:** Laboratory tests findings in SSc patients with and without myocardial involvement

	Myocardial involvement (n = 21)	Non- myocardial involvement (n = 63)	P value
Leukopenia	0 (0)	1 (1.6)	1
Thrombocytopenia	2 (9.5)	5 (7.9)	1.000
Elevated IgG	8 (38.1)	22 (34.9)	0.793
Elevated IgA	1 (4.8)	9 (14.3)	0.437
Elevated IgM	1 (9.5)	6 (9.5)	1
Decreased C3	0 (0)/20	7 (11.3)/72	0.186
Decreased C4	1 (5)/20	5 (8.1)/62	1
Elevated ESR	6 (28.6)	23 (37.1)/62	0.479
HsCRP (mg/L)	2.9 (1.4,9.8)	1.5 (0.7,3.7)/61	0.022
Elevated CK	7 (33.3)	3 (4.8)	0.002
ANA positivity	21 (100)	56 (88.9)	0.254
Anti-RNP antibody positivity	4/20 (20)	20 (31.7)	0.313
Anti SCL70 antibody positivity	15 (71.4)	23 (36.3)	0.005
ACA positivity	2/20 (10)	6/59 (10.2)	1
Anti PM-Scl antibody positivity	1/19 (5.3)	1/56 (1.8)	0.445

Ig: immunoglobulin; C3/4: Complement 3/4; ESR: erythrocyte sedimentation rate; hsCRP: High-sensitivity C-reactive protein; CK: creatine kinase; ANA: antinuclear antibody; ACA: anticentromere antibody; RNP: U1-ribonucleoprotein; Anti Scl-70: anti-topoisomerase; PM-Scl: polymyositis-scleroderma

### Clinical characteristics of myocardial involvement

The mean age at the time of MI diagnosis was 46.6 ± 15.8 years and the median duration from the onset of SSc to MI was 22.0 (3.5, 70.5) months. Of the 21 patients with MI, 14 (66.7%) presented with cardiovascular symptoms, including 9 (42.8%) chest distress, 4 (19%) palpitations, 11 (52.4%) shortness of breath after exercise. The detailed data in each patient are listed in Table 4. No significant differences were found between SSc patients with clinical and subclinical MI. (Table 5). Of the patients with MI, 15 (71.4%) underwent CMR and 12 had myocardial fibrosis; 4 of these 12 patients showed a LVEF < 50% on MRI. The sites of myocardial fibrosis included the left ventricle (10 cases), the right ventricle (2 cases) and the interventricular septum (4 cases); 4 cases had multiple sites of fibrosis simultaneously. Myocardial ischemia was detected in 4 cases. CMR revealed myocardial inflammation in one case.

**Table 4:** Cardiac findings in SSc patients with myocardial involvement

Patient	Age of MI (years)	Duration of MI (months)	Heart failure	Elevated CTni	Elevated NT-proBNP	ECG	Echo	CMR
1	14	2	Y	Y	Y	Ventricular arrhythmia	Cardiomyopathy	LGE +
2	21	2	Y	Y	Y	-	LVEF < 50%	LGE +
3	29	27	Y	Y	Y	Atrioventricular block	Wall motion abnormality LVEF < 50%	LGE + Ventricular hypertrophy
4	34	34	Y	Y	Y	-	LVEF < 50%	NA
5	34	124	N	Y	Y	Ventricular arrhythmia	Cardiomyopathy Ventricular hypertrophy	Ventricular hypertrophy
6	35	80	Y	Y	Y	Ventricular hypertrophy	Cardiomyopathy LVEF < 50%	NA
7	35	7	Y	Y	Y	-	Wall motion abnormality LVEF < 50%	NA
8	37	28	N	Y	N	-	Ventricular hypertrophy	NA
9	38	37	Y	N	Y	Supraventricular arrhythmia	Wall motion abnormality	LGE +
10	45	0	Y	N	Y	-	Cardiomyopathy LVEF < 50%	LGE +
11	44	12	Y	Y	Y	Atrioventricular block	Cardiomyopathy LVEF < 50%	Wall motion abnormality
12	52	61	N	N	N	Ventricular arrhythmia	-	LGE +
13	51	2	N	N	N	-	-	LGE + Ventricular hypertrophy
14	55	13	N	NA	Y	-	Cardiomyopathy Ventricular hypertrophy LVEF < 50%	NA
15	55	108	N	Y	N	-	-	LGE + Ventricular hypertrophy
16	60	130	Y	Y	Y	Ventricular arrhythmia	-	LGE +
17	60	5	Y	Y	Y	Atrioventricular block	LVEF < 50%	NA
18	62	205	N	N	Y	-	Wall motion abnormality	LGE + Ventricular hypertrophy
19	64	2	N	Y	Y	-	Ventricular enlargement	Ventricular enlargement
20	68	55	N	NA	Y	-	LVEF < 50%	LGE + Ventricular hypertrophy
21	69	20	N	Y	Y	Atrial fibrillation	-	LGE + Ventricular hypertrophy

CTnI: cardiac troponin I; NTproBNP: N-terminal pro-brain natriuretic peptide; CMR: cardiac magnetic resonance; LV: left ventricular; EF: Ejection fraction; LGE: late gadolinium enhancement; NA: not available; Y: yes; N: No; -: normal

**Table 5:** Characteristics of the SSc patients with clinical and subclinical myocardial involvement

	Clinical involvement(n = 14)	Subclinical involvement(n = 7)	P
Immunological characteristics			
Elevated ESR	2 (14.3)	4 (57.1)	0.120
Elevated CTnI	11 (78.6)	3/5 (60)	0.570
Elevated NT-proBNP	11 (78.6)	6 (85.7)	1.000
UCG and ECG abnormalities			
Ventricular arrhythmia	3 (21.4)	1 (14.3)	1.000
Supraventricular arrhythmia	1 (7.1)	1 (14.3)	1.000
Atrioventricular block	2 (14.3)	0 (0)	0.533
Atrial fibrillation	1 (7.1)	0 (0)	1.000
RV systolic dysfunction	5 (35.7)	1 (14.3)	0.613
LV diastolic dysfunction	4 (28.6)	2 (28.6)	1.000
LVEF < 50%	6 (42.9)	4 (57.1)	0.659
Pericardial effusion	7 (50)	2 (28.6)	0.642
CMR abnormalities			
LGE positivity	8/10 (80)	4/5 (80)	1.000

LV: left ventricular; RV: right ventricular; EF: Ejection fraction; ESR: Erythrocyte sedimentation rate; NT-proBNP: N-terminal pro-B-type natriuretic peptide; cTnI: cardiac troponin I; CMR: cardiac magnetic resonance; LGE: late gadolinium enhancement

### Treatment and outcomes

Nineteen (90.5%) SSc patients with MI were treated with both corticosteroid and immunosuppressants. 2 patients did not use corticosteroid or immunosuppressants. Four of them (19%) received glucocorticoid pulse therapy due to progressed myocardial involvement. One patient received an increased dose of prednisone (from 0.2 mg/kg/day to 1 mg/kg/day) due to the new occurrence of arrhythmia. The remaining patients continued the former dose of glucocorticoid even if MI was detected. The number of patients who were treated with cyclophosphamide, methotrexate, mycophenolate mofetil, and leflunomide were 18 (85.7%), 2 (9.5%), 1 (4.8%) and 1 (4.8%) respectively. Symptomatic treatments for heart disease, including angiotensin-converting enzyme inhibitors/angiotension II receptor blockers (ACEi/ARB), beta -blockers, diuretic, sacubitril/valsartan were used to control ventricular rate, reduce load and improve ventricular remodeling in 17 (81%) patients.

Eleven (52.4%) patients were followed-up for a median period of 15.5 (7.8, 29.5) months. Four (36.4%) patients developed new abnormalities in the LVEF (from > 50% to < 50% on echocardiography). Six (54.5%) patients underwent repeated CMR tests but no improvement was found on myocardial involvement (Table 6). In particular, we have also focused on the prognosis of patients who received glucocorticoid pulse therapy. Two patients with pulse therapy were followed up for 13.4 months and 10 months respectively. LVEF by echocardiography did not improve significantly in either of two patients. Levels of CTnI both decreased but still higher than normal in one patient.

**Table 6:** The characteristics of cardiac magnetic resonance of the patients before and after treatment

No.	Immunosuppressive treatment	Interval between two CMR test(months)	Before treatment				After treatment			
			LV EF (%)	RV EF (%)	Hypoperfusion/location	Gadolinium/location	LV EF (%)	RV EF (%)	Hypoperfusion/location	Gadolinium/location
1	CTX + G	5.7	51.2	42.9	LV inferior	LV + RV diffuse subendocardial	38.6	19.9	Ventricle septal + LV	LV + RV diffuse subendocardial
2	MMF + G	16.2	51.8	35.5		LV apical	44.4	30.5		LV apical
3	CTX + G	6.3	31.5	22.8		LV + RV subendocardial	26.4	22.5		LV + RV subendocardial
4	CTX + G	5.9	77.7	68.2		Ventricular septal + LV	67.6	62.4		Ventricular septal + LV
5	N	32.9	57	60.6		Ventricular septal	56.3	47.7		Ventricular septal
6	CTX + G	26.4	42.4	50.6	LV lateral	Ventricular septal + LV	43.7	48.4	LV lateral	Ventricular septal + LV

G: glucocorticoid; CTX: cyclophosphamide; MMF: mycophenolate; N: no; LV: left ventricular; RV: right ventricular; EF: Ejection fraction

## Discussion

Systemic sclerosis is a complex multi-systemic disease. Due to the different definitions used in previous studies, the incidence of myocardial involvement ranges widely from 15 to 66.7% [7, 8] with most cases exhibiting insidious progression. A literature review and meta-analysis on the survival of patients with SSc showed that 19% of deaths were caused by heart disease, and that cardiac involvement significantly increased the risk of death [9]. The pathogenesis of MI in SSc remains unclear although multiple factors have been described previously, including repeated ischemia-reperfusion injury of the heart, vascular microcirculation disorders and inflammatory reactions. Irreversible focal fibrosis is known to lead to heart failure and arrhythmias [10–14]. In some autopsy studies, myocardial fibrosis was found to be very common in SSc patients, with an incidence of up to 81% [10, 15].

Previous studies which focused on SSc with cardiac involvement have shown that SSc patients with heart involvement had an older onset age of SSc [16], more common in lcSSc [17], and fibrosis of the heart was correlated with a higher modified Rodnan skin score (mRSS) in subclinical cardiac involvement [18]. But these studies focused more on all kinds of cardiac involvements, including structural cardiac abnormalities, arrhythmia, heart failure and decreased global myocardial perfusion, etc. Our study only focused on existing myocardial involvement which is one part of cardiac alterations, and different study objects may lead to different results. Similar to cardiac involvement, patients with MI were older at the onset of SSc, but dcSSc was more common numerically, and with a higher frequency of anti-Scl70 antibody positivity. A previous study showed myocardial involvement was more common and more severe in dcSSc [15]. Therefore, we should remain vigilant for MI in patients with anti-ScL70 antibody positivity and wide skin sclerosis. Our study showed that patients with MI commonly exhibited CK elevation and myositis. Myositis mainly involves the skeletal muscle but also occasionally affects the myocardium. Aggressive immunosuppressive treatment may control myocardial inflammation and improve cardiac function in patients with inflammatory cardiomyopathy. However, the efficacy of immunosuppressive treatment for MI in SSc patients is still not determined. A recent study found that subclinical cardiac fibrosis in SSc was associated with increase of highly sensitive troponin I (cTNI) and N-terminal brain natriuretic peptide precursor (NT-proBNP) [19]. In the present study, 14/19 patients showed elevated levels of cTnI and 17/21 patients showed elevated levels of NT-proBNP, may be suggesting the importance of these two biomarkers in predicting MI in patients with SSc. Echocardiography is widely used to evaluate cardiac structure but has low sensitivity for detecting myocardial fibrosis in the early stages. Recently, it has been reported that the calculation of stress by the speckle tracking echocardiographic techniques can identify impaired systolic function of both ventricles in patients with no obvious cardiac symptoms and a normal left ventricular ejection fraction [20]. Late gadolinium enhancement (LGE) CMR is considered to be the non-invasive gold standard for imaging macroscopic myocardial fibrosis. A previous study showed that CMR can identify initial cardiac injury in SSc patients earlier than echocardiography and could detect abnormalities prior to the onset of clinical symptoms [21, 22]. In a prospective study of 201 patients with SSc [23], late gadolinium enhancement (LGE) with negative T2-weighted images was detected in 27.9% patients without known SSc-related cardiac involvement by CMR. However, this technique is not widely used in SSc patients due to limitations imposed by high costs, long appointment times, long scan times, allergies and the deterioration of renal function arising from the use of contrast agents. In conclusion, regular annual monitoring echocardiography is recommended for all patients with SSc while CMR is recommended for those suspected of having myocardial involvement.

There is still no effective treatment for MI in SSc patients. It is unknown whether immunosuppressive therapy could prevent or cure myocardial lesions in this population. Theoretically, immunotherapy should be helpful to control myocardial inflammation although it is still difficult to accurately determine the presence of myocardial inflammation. De Luca proposed the concept of targeted treatment with an interleukin-1 antagonist; however, this strategy has not been described further in the existing literature [24]. In addition to immunotherapy, a previous study showed that some treatments with potent vasodilator activity on small coronary arteries, such as dipyridamole, may be beneficial in the treatment of SSc patients with myocardial perfusion abnormalities [25]. One patient in our study showed a gradual decrease of LVEF (58% down to 47%) 4 months after the discontinuation of sacubitril/valsartan, but it improved again (60%) in 2 months after re-treatment with sacubitril/valsartan without the adjustment of other treatments. The results of the DeSScipher cohort study also showed that vasodilators, in particular calcium channel blocker (CCB), and/or angiotensin-converting enzyme inhibitors (ACEi), and/or angiotension II receptor blockers (ARB) and low-dose acetylsalicylic acid reduced the occurrence of different types of myocardial involvement [26].

There are some limitations in this study. Firstly, due to the lack of literature consensus on the definition of MI [6, 27, 28], we defined MI based on imaging (UCG or CMR). Due to the retrospective nature of our study, all patients underwent echocardiography, which is not sensitive enough for identification of early stage MI, while only part of patients with abnormal heart manifestations underwent further CMR evaluation. Thus, subclinical myocardial damage may have been missed in some patients. Secondly, this is a single-center study with only a small number of patients, and some patients did not have follow-up. We did not analyze the long-term prognosis in patients with different immunotherapy. The efficacy of immunosuppressive treatment for MI in SSc patients is still not determined. Future prospective study with larger sample size should be done.

## Conclusion

Myocardial involvement is common in systemic sclerosis. Effect of immunotherapy is still unknown for myocardial involvement in systemic sclerosis. Regular monitoring of CTnI, NT-proBNP and echocardiography may be helpful for earlier diagnosis of MI. For SSc patients with a high suspicion of MI, CMR should be considered especially in patients with normal UCG.

## Data Availability

Not applicable.

## List of Abbreviations

SSc: Systemic sclerosis
CMR: cardiac magnetic resonance
UCG: echocardiography
MI: myocardial involvement
dcSSc: diffuse cutaneous SSc
lcSSc: limited cutaneous SSc
LVEF: left ventricular ejection fraction
ILD: Interstitial lung disease
HRCT: high-resolution computed tomographic
PAH: Pulmonary arterial hypertension
mPAP: pulmonary arterial pressure
PAWP: pulmonary artery wedge pressure
PVR: pulmonary vascular resistance
RHC: right heart catheterization
SPAP: systolic pulmonary artery pressure
LGE: late gadolinium enhancement
TATE: octreotate
FAPI: fibroblast activation protein inhibitor
ESR: Erythrocyte sedimentation rate
cTnI: cardiac troponin I
NT-proBNP: N-terminal pro-B-type natriuretic peptide
CCB: calcium channel blocker
ACEi: angiotensin-converting enzyme inhibitors
ARB: angiotension II receptor blockers
